# Contribution of socioeconomic status to the risk of small for gestational age infants – a population-based study of 1,390,165 singleton live births in Finland

**DOI:** 10.1186/1475-9276-12-28

**Published:** 2013-05-01

**Authors:** Sari Räisänen, Mika Gissler, Ulla Sankilampi, Juho Saari, Michael R Kramer, Seppo Heinonen

**Affiliations:** 1 Department of Obstetrics and Gynaecology, Kuopio University Hospital, Kuopio, Finland; 2 National Institute for Health and Welfare (THL), Helsinki, Finland; 3 Nordic School of Public Health, Gothenburg, Sweden; 4 Department of Pediatrics, Kuopio University Hospital, Kuopio, Finland; 5 Kuopio Welfare Research Centre (KWRC) and Department of Social Sciences, The University of Eastern Finland, Kuopio, Finland; 6 Department of Epidemiology, Rollins School of Public Health, Emory University, Atlanta, GA, USA; 7 Clinical Medicine, University of Eastern Finland, Kuopio, Finland

## Abstract

**Background:**

Small for gestational age (SGA) infants are at increased risk of short- and long-term adverse outcomes.

**Methods:**

Population-based case–control study using data derived from the Finnish Medical Birth Register for the years 1987–2010 (total population of singleton live births *n* = 1,390,165). The aim was to quantify the importance of risk factors for SGA and describe their contribution to socioeconomic status (SES) disparities in SGA by using logistic regression analysis.

**Results:**

Of all the singleton live births (*n* = 1,390,165), 3.1% (n = 42,702) were classified as SGA (defined as below 2 standard deviations of the sex-specific population reference mean for gestational age). The risk of SGA was 11 − 24% higher in the lower SES groups compared to the highest SES group. Smoking alone made the largest contribution, explaining 41.7 − 50.9% of SES disparities in SGA. The risk of SGA was 2.3-fold and 7% higher among women who smoked or had quit smoking during the first trimester of pregnancy (adjusted odds ratio (aOR) 2.34, 95% CI 2.28-2.42 and aOR 1.07, 95% CI 1.00 − 1.15, respectively) compared with the non-smokers.

**Conclusions:**

SGA is substantially affected by SES. Smoking explained up to 50% of the difference in risk of SGA between high and low SES groups. Quitting smoking during the first trimester of pregnancy resulted in a 7% higher incidence of SGA comparable to that of non-smoking women. Thus, interventional attempts to reduce smoking during pregnancy might help to decrease the socioeconomic gradient of SGA.

## Background

Small for gestational age (SGA) infants are at increased risk of perinatal morbidity and mortality [1,2] as well as long-term adverse health [3] and developmental outcomes [4]. Therefore, it is important to recognize the potential risk factors for SGA during pregnancy so that preventive measures can be targeted at the most at risk subgroups of pregnant women. Fetal growth restriction (FGR) is multifactorial and caused by maternal, fetal or placental factors. Maternal factors include demographic variables and medical disorders, e.g., maternal age [5,6], nulliparity [6,7], low maternal body mass index (BMI) [5], high maternal BMI [5], short maternal stature [7], hypertension [6,7] and preeclampsia [6-8]. Birthweight and risk of SGA have been shown to vary with socioeconomic status (SES) in multifarious ways [9], even in countries with a welfare system, such as Finland [10,11]. Maternal smoking is more prevalent among low SES groups [10,12] and is also a well-known risk factor for adverse birth outcomes such as SGA [10,12,13].

The aim of the present study was to identify risk factors for SGA among singleton live births and quantify their contribution to socioeconomic disparities in SGA using data for the total population of singleton live births (n = 1,390,165) during the years 1987–2010 in Finland (with around 5.5 million residents and mainly publicly funded health services). In previous studies, SGA has typically been defined as an infant who is below the tenth percentile of length and weight for their gestational age [14]. However, in the present study, the definition of SGA was a birthweight more than two standard deviations (SDs) below the mean weight for the gestational age which selected the cases more strictly than the 10th percentile and therefore the most serious cases only were analyzed.

## Methods

### Data and population

Data were extracted from the Finnish Medical Birth Register (MBR), which is a compilation of the clinical records of all of the obstetric care units in Finland. The MBR is currently maintained by the National Institute for Health and Welfare (THL), which authorized the use of sensitive health register data in scientific research for the years 1987–2010, as required by national data protection legislation.

The MBR includes information on maternal and neonatal birth characteristics and perinatal outcomes (all live births or stillbirths delivered after the 22nd gestational week or weighing 500 g or more). For each infant, an electronic form (or rarely a paper form) has to be filled in by the hospital with information on the newborn during the first seven days of life. The delivery units and register controller actively collaborate to guarantee the validity of the data. For example, information submitted to the MBR is checked, and any missing or inferred to be incorrect information is queried with the treating hospitals before correcting. Some newborns (less than 0.1%) are missing from the MBR, and therefore it was supplemented with data compiled by the Population Register Centre on live births and data compiled by Statistics Finland on stillbirths and deaths during the first week of life. After these additions, the data covered 100% of birth events. The data included information on all singleton live births (*n* = 1,390,165) in Finland for the years 1987–2010.

### Ethical approval

Permission to use the confidential register data in this study was granted on February 16^th^, 2012 by the National Institute for Health and Welfare (THL) in Finland. (Reference number 1749/5.05.00/2011). Informed consent of the registered individuals was not needed since only anonymized data from national health registers were used, and the individuals were not contacted.

### Variables and definitions

SGA was defined according to the International Societies of Pediatric Endocrinology and the Growth Hormone Research Society as a birth weight more than 2 SDs below the sex- and gestational age-specific reference mean [15]. The Finnish population-based reference for birth size was used to classify the newborn infants as SGA (Unpublished Finnish data from THL). Gestational age was estimated based on parturient’s last menstrual period, unless there was a discrepancy with the first- or second-trimester ultrasonography measurements of more than seven or 14 days, respectively, in which case the latter measurements were used. Information on gestational age and birthweight was missing in 8,754 (0.6%) and 1,304 (0.1%) cases, respectively, and therefore those cases were excluded from the analyses. Information on in-vitro fertilization (IVF) was only available from 1991 onwards. However, only 170 IVF children were born in Finland before 1991. IVF also included intracellular sperm injection (ICSI) and frozen embryo transfers (FET). Maternal anemia was defined as haemoglobin levels below 100 g/L. Maternal smoking was self-reported and recorded as non-smoking or smoking during pregnancy. After 1991, information was also available about the number of women who quitted smoking during the first trimester or continued smoking throughout the pregnancy. Information on maternal height and weight was available from 2004 onwards, and thus multivariate analyses of the body mass index (BMI) were only performed for the years 2004–2010. BMI was calculated by dividing body weight in kilograms by the squared height in meters (kg/m^2^). Information on socioeconomic status (SES) based on mother’s occupation at time of birth was gathered in the register from October 1990, and thus used in the analyses for the years 1991–2010. SES was recorded as either upper white-collar workers such as teachers and physicians, lower white-collar workers such as nurses and secretaries, blue-collar workers such as cookers and cleaners or “others”, as described in detail elsewhere [10]. The category “others” included entrepreneurs, housewives, students, retired, unemployed and unclassifiable women. Information on SES was missing in 151,694 (13.2%) cases, and hence they were analyzed as a separate group in the multivariate analyses. Parturients’ place of residence was grouped by the twenty hospital districts in Finland. Ethnic variation is Finnish population was minor during the study period [16]. Marital status was recorded as married or cohabiting, or single. The study period from 1987–2010 was divided into five time periods (1987–1991, 1992–1996, 1997–2001, 2002–2006, 2007–2010) to examine the variation in SGA incidence over time.

Information on major congenital anomalies was obtained from the Finnish Register of Congenital Malformations established in 1963 and currently maintained by THL. The two data sources were linked together using encrypted unique personal identification numbers. Apart from number of deliveries, miscarriages and prior terminations, the data were dichotomous or categorical.

### Statistical analyses

Statistical differences in frequencies (categorical and dichotomous variables) between women with SGA infants and the reference population were evaluated by Pearson’s chi-square test. Differences between continuous variables were evaluated by Student’s t- and Mann–Whitney U-tests as appropriate. Multivariate logistic regression analyses were used to model the risk factors for SGA in comparison with controls in 1991–2010 due to availability of information on SES and smoking status. Odds ratios (OR) with 95% confidence intervals (CI) were calculated. Possible confounding variables were selected based on univariate analyses (*p* < 0.05). Differences were deemed to be significant if *p* < 0.05. Furthermore, to quantify the contribution of SGA risk factors (smoking, parturient’s place of residence grouped by hospital district, amniocentesis, placenta previa, placental abruption, congenital anomaly and prior cesarean section (CS)) to socioeconomic disparities, we evaluated the percentage reduction in the SES – SGA association in sequential logistic regression models. Each covariate was added separately to a partially adjusted model (Model 3: adjusted for smoking, SES, maternal age, and parity), and the percentage reduction in the odds ratio was calculated. The formula used was (OR Model 3 – OR Model A/B/C/D) / (OR Model 3 – 1) [17]. The data were analyzed using SPSS for Windows 19.0, Chicago, IL.

## Results

The study population included all singleton live births (*n* = 1,390,165) for the years 1987–2010, of which 42,702 (3.1%) were classed as SGA. Women with SGA infants were significantly more likely to be nulliparous, smokers and deliver a male fetus by cesarean section at lower gestational age compared with the controls (Tables 1 and 2). Furthermore, women with an SGA infant exhibited significantly more reproductive risk factors, such as placenta previa, placental abruption and congenital anomalies and underwent IVF, induction, chorionic villus biopsy and amniocentesis significantly more often than the controls. There were significant socioeconomic differences between the groups; non-SGA infants were relatively over-represented among the upper white-collar workers, whereas SGA infants were over-represented among blue-collar workers. All deliveries were grouped by parturient’s place of residence and hospital districts as shown in Table 3. The incidence of SGA varied from 2.4% to 3.8% among the 20 hospital districts in Finland (*p* ≤ 0.001).

**Table 1:** **Pregnancy and delivery characteristics of women with singleton live births (*n*** **= 1,390,165) classified as SGA/not SGA (analyzed by chi-square and Mann–Whitney U tests)**

**Characteristic% or Mean (±SD)**	**SGA, *n*** **= 42,702 (3.1%)**	**Not SGA, *n*** **= 1,347,463**	***p *value**
Mean maternal age (±SD) (years)	29.0 (±5.8)	29.1 (±5.3)	≤0.001
≤19	4.2	2.7	≤0.001
20 − 29	50.5	51.5	
30 − 39	41.2	42.8	
≥40	4.1	3.0	
Primiparous women	59.6	40.6	≤0.001
Multiparous women	40.4	59.4	
Mean gestational age (weeks)	38.5 (±3.1)	39.8 (±1.7)	≤0.001
<28	1.9	0.2	
28 − 31 + 6	2.9	0.4	
32 − 36 + 6	12.7	3.5	
37 − 39 + 6	47.1	42.3	
40 − 41 + 6	32.5	48.8	
>42	2.9	4.8	
*Mode of delivery*			
Vaginal delivery	63.0	78.8	≤0.001
Breech	1.0	0.4	
Forceps	0.1	0.1	
Vacuum assistance	5.4	5.9	
Cesarean section	30.5	14.7	
Girl	48.5	48.9	0.17
Boy	51.5	51.1	
Mean birthweight (±SD) (grams)	2372.3 (±518.2)	3590.4 (±514.4)	≤0.001
Region (n = 20) range in 1987 − 2010	3.1 (2.9 − 3.4)	96.9 (96.6 − 97.1)	≤0.001
Time periods			
1987 − 1991	3.0	97.0	≤0.001
1992 − 1996	2.9	97.1	
1997 − 2001	3.1	96.9	
2002 − 2006	3.1	96.9	
2007 − 2010	3.3	96.7	

SD = standard deviation.

**Table 2:** **Reproductive risk factors of small for gestational age (SGA) among the total population of singleton live births (*n*** **= 1,390,165) for the years 1987–2010 in Finland**

**Characteristic% or Mean (±SD)**	**SGA, *n*** **= 42,702 (3.1%)**	**Not SGA, *n*** **= 1,347,463**	***p *value**
Pregravid BMI^a^			≤0.001
≤24.9	72.8	66.9	
25–29.9	17.8	21.6	
≥30	9.4	11.5	
Non-smoking	71.1	84.9	≤0.001
Quitted smoking	4.5	3.5	
Smoking	24.3	11.6	
Married or cohabiting	93.7	95.9	≤0.001
Mean number of deliveries (±SD)	0.71 (±1.22)	1.04 (±1.35)	≤0.001
Mean number of pregnancies (±SD)	1.18 (±1.63)	1.48 (±1.70)	≤0.001
Mean number of miscarriages (±SD)	0.26 (±0.65)	0.26 (±0.61)	0.72
Mean number of prior terminations (±SD)	0.14 (±0.45)	0.12 (±0.41)	≤0.001
IVF^b^	1.3	1.1	0.001
Anemia ≤ 100 g/l	0.6	0.6	0.28
Prior Cesarean section	7.6	8.4	≤0.001
Chorionic villus biopsy	1.1	0.9	≤0.001
Amniocentesis	3.9	2.7	≤0.001
Placenta previa	0.3	0.2	≤0.001
Placental abruption	0.6	0.2	≤0.001
Induction	18.8	13.3	≤0.001
Congenital anomalies	8.7	2.8	≤0.001
Socioeconomic status			
Upper white-collar worker	5.5	6.7	≤0.001
Lower white-collar worker	31.2	32.6	
Blue-collar worker	15.9	13.6	
Other	19.5	19.8	
Missing	27.9	27.2	

SD = standard deviation, ^a^BMI = body mass index, data cover the years 2004–2010.

*n* = 366,542, ^b^IVF = in vitro fertilization, data cover the years 1991–2010.

**Table 3:** Prevalence of SGA infants among all singleton live births grouped by place of residence and hospital district for the years 1987–2010 in Finland

**Number**	**Hospital district**	**Number of births, *n***	**% Prevalence of SGA (range in 1987–2010)**	***p *value**
1	South Karelia	31,106	3.3 (2.6 − 3.6)	≤0.001
2	South Ostrobothnia	53,413	2.4 (2.2 − 2.7)	
3	South Savo	25,525	3.1 (3.0 − 3.6)	
4	Helsinki and Uusimaa	397,593	3.3 (3.2 − 3.4)	
5	East Savo	10,384	3.7 (2.8 − 4.3)	
6	Kainuu	20,602	3.4 (3.0 − 4.0)	
7	Kanta-Häme	42,501	3.2 (2.9 − 3.4)	
8	Middle Ostrobothnia	21,987	2.7 (2.4 − 3.6)	
9	Central Finland	69,985	2.9 (2.6 − 3.2)	
10	Kymenlaakso	41,486	3.2 (2.9 − 3.9)	
11	Lapland	32,010	3.8 (3.5 − 4.8)	
12	Länsi-Pohja	17,950	3.2 (2.5 − 3.3)	
13	Pirkanmaa	115,602	2.8 (2.5 − 2.9)	
14	North Karelia	43,296	3.2 (2.9 − 3.7)	
15	Northern Ostrobothnia	121,927	3.1 (2.9 − 3.3)	
16	North Savo	62,892	3.4 (3.1 − 3.9)	
17	Päijät-Häme	51,397	3.2 (2.7 − 3.9)	
18	Satakunta	60,444	2.8 (2.4 − 3.3)	
19	Vaasa	42,718	2.4 (2.2 − 2.9)	
20	Varsinais-Suomi	112,131	2.7 (2.3 − 3.2)	
	Total	1,374,458	3.1 (2.9 − 3.4)	

After adjustment for background information, the OR data suggested that factors placing women at a high risk (aOR >2.0) of SGA were primiparity, smoking and congenital anomaly, whereas risk factors placing women at a moderate risk (aOR <2.0) of SGA were an advanced maternal age, BMI ≤ 24.9, amniocentesis, placental abruption, prior CS, single marital status and low SES (Table 4). Lower white-collar workers and blue-collar workers had 11% (95% CI 6 − 17%) and 24% (95% CI 17 − 30%) higher risk of SGA, respectively, in comparison with the upper white-collar workers. The risk of SGA varied significantly in 15 of the 20 hospital districts in comparison with hospital district 2 with the lowest SGA rate (2.4%). After adjustment, an up to 1.54-fold difference in SGA risk was detected between hospital districts.

**Table 4:** **Unadjusted and adjusted odds ratios (ORs) of SGA (*n *= 30,443) among the total population of singleton live births (*n *= 1,007,977) for the years 1991–2010 in Finland**

**Characteristic/risk factors**	**Unadjusted OR**	**Adjusted OR**
Maternal age (years)		
≤19	1	1
20-29	0.63 (0.60–0.67)	1.08 (1.04–1.18)**
30-39	0.62 (0.59–0.65)	1.37 (1.28–1.46)***
≥40	0.87 (0.81–0.94)	1.82 (1.66–1.99)***
Primiparity	2.20 (2.15–2.24)	2.41 (2.33-2.48)***
Gestational age (weeks)		
<28	7.30 (6.70–7.95)	6.26 (5.65–6.93)***
28 − 31 + 6	7.35 (6.85–7.89)	5.90 (5.43–6.40)***
32 − 36 + 6	3.17 (3.06–3.28)	2.74 (2.63–2.84)***
37 − 39 + 6	1	1
40 − 41 + 6	0.59 (0.58–0.61)	0.59 (0.58–0.61)***
>42	0.51 (0.47–0.54)	0.44 (0.41–0.47)***
Pregravid BMI^a^		
≤24.9	1.32 (1.24–1.41)	1.38 (1.28–1.48)***
25-29.9	1.00 (0.93–1.08)	1.06 (0.98–1.15)
≥30	1	1
Smoking status		
Non-smoking	1	1
Quitted smoking	1.28 (1.20 − 1.36)	1.07 (1.00 − 1.15)*
Smoking	2.45 (2.39–2.51)	2.34 (2.28–2.42)***
Single marital status	1.58 (1.51–1.64)	1.09 (1.04–1.15)***
Socioeconomic status (SES)		
Upper white-collar workers	1	1
Lower white-collar workers	1.16 (1.11–1.21)	1.11 (1.06–1.17)***
Blue-collar workers	1.42 (1.35–1.49)	1.24 (1.17–1.30)***
Other	1.19 (1.14–1.25)	1.13 (1.07–1.19)***
Missing	1.36 (1.29 − 1.43)	1.15 (1.08 − 1.21)***
Prior pregnancies (number)	0.88 (0.87–0.89)	0.98 (0.97–0.99)***
Prior terminations (number)	1.11 (1.08–1.13)	1.00 (0.97–1.03)
IVF^b^	1.19 (1.07–1.32)	0.83 (0.74–0.92)***
Chorionic villus biopsy	1.22 (1.11–1.34)	1.08 (0.97–1.21)
Amniocentesis	1.44 (1.37–1.51)	1.30 (1.23–1.38)***
Placenta previa	1.47 (1.23–1.75)	0.75 (0.61–0.91)**
Placental abruption	3.36 (2.94–3.83)	1.41 (1.22–1.64)***
Major congenital anomaly	3.19 (3.08–3.32)	2.51 (2.40–2.62)***
Prior Cesarean section	0.89 (0.86–0.93)	1.26 (1.21–1.29)***
Time periods		
1991 − 1996	1	1
1997 − 2001	1.05 (1.02 − 1.08)	1.04 (1.00 − 1.07)*
2002 − 2006	1.07 (1.04 − 1.10)	1.08 (1.04 − 1.11)***
2007 − 2010	1.14 (1.11 − 1.18)	1.17 (1.13 − 1.21)***
Place of residence grouped by hospital districts		
1	1.40 (1.27 − 1.53)	1.29 (1.17 − 1.42)***
2	1	1
3	1.29 (1.17 − 1.43)	1.20 (1.08 − 1.34)***
4	1.38 (1.29 − 1.47)	1.21 (1.13 − 1.30)***
5	1.64 (1.44 − 1.86)	1.51 (1.32 − 1.73)***
6	1.45 (1.31 − 1.61)	1.38 (1.24 − 1.54)***
7	1.36 (1.25 − 1.48)	1.18 (1.08 − 1.30)***
8	1.13 (1.02 − 1.26)	1.17 (1.05 − 1.32)**
9	1.19 (1.10 − 1.28)	1.11 (1.02 − 1.21)*
10	1.35 (1.24 − 1.47)	1.21(1.10 − 1.32)***
11	1.66 (1.52 − 1.81)	1.54 (1.40 − 1.70)***
12	1.31 (1.17 − 1.46)	1.13 (1.00 − 1.28)*
13	1.14 (1.06 − 1.23)	1.05 (0.97 − 1.14)
14	1.33 (1.22 − 1.45)	1.27 (1.16 − 1.40)***
15	1.31 (1.22 − 1.54)	1.32 (1.22 − 1.42)***
16	1.43 (1.32 − 1.54)	1.34 (1.23 − 1.45)***
17	1.35 (1.25 − 1.47)	1.21(1.11 − 1.32)***
18	1.15 (1.06 − 1.25)	1.04 (0.96 − 1.14)
19	1.02 (0.93 − 1.11)	1.00 (0.91 − 1.10)
20	1.15 (1.07 − 1.23)	0.97 (0.89 − 1.05)

SGA = small for gestational age, ^a^Body mass index (BMI), data cover the years 2004–2010, *p ≤ 0.05, **p ≤ 0.01, ***p ≤ 0.001.

We quantified the contribution of each risk factor for SGA (smoking, parturient’s place of residence grouped by hospital district, amniocentesis, placenta previa, placental abruption, major congenital anomaly and prior CS) to socioeconomic disparities by using logistic regression and calculating the percentage reduction in the OR (Table 5, only contributions above zero are presented). Model 1 described the OR of SGA adjusted by SES alone. After smoking was added to Model 2 (adjusted for SES, age and parity), the OR of SGA decreased across the SES stratum, and smoking alone explained 41.7 − 50.9% of the difference in SGA risk between SES groups. The contributions of placenta previa, placental abruption and prior CS to SGA incidence were null between all groups. Model C shows that amniocentesis, placental abruption, placenta previa, congenital anomaly and prior CS altogether explained 3.6% of the excess SGA risk among the lowest SES groups. Further, maternal age and parity did not contribute to the difference in risk of SGA between SES groups.

**Table 5:** Odds ratios (ORs) of socioeconomic status (SES) for SGA after adjustments for maternal and pregnancy characteristics and other significant risk factors for SGA

												
	**Model 1**	**Model 2**	**Model 3**		**Model A**		**Model B**		**Model C**		**Model D**	
**SES**	**OR (95% CI)**	**OR (95% CI)**	**OR (95% CI)**	Diff. with 2 (%)*	**OR (95% CI)**	Diff. with 3 (%)*	**OR (95% CI)**	Diff. with 3 (%)*	**OR (95% CI)**	Diff. with 3 (%)*	**OR (95% CI)**	Diff. with 3 (%)*
Upper white-collar	1	1	1		1		1		1		1	
Lower white-collar	1.16 (1.11 − 1.21)	1.24 (1.18 − 1.29)	1.14 (1.09 − 1.20)	41.7	1.14 (1.09 − 1.19)	-	1.14 (1.09 − 1.20)	-	1.14 (1.09 − 1.19)	-	1.14 (1.09 − 1.19)	-
Blue-collar	1.42 (1.35 − 1.48)	1.57 (1.49 − 1.64)	1.28(1.21 − 1.34)	50.9	1.28 (1.22 − 1.35)	-	1.27 (1.21 − 1.34)	3.6	1.27 (1.21 − 1.34)	3.6	1.27 (1.21 − 1.33)	3.6
Other	1.19 (1.14 − 1.25)	1.28 (1.22 − 1.34)	1.19 (1.14 − 1.25)	32.1	1.18 (1.13 − 1.24)	5.3	1.18 (1.13 − 1.24)	5.3	1.19 (1.13 − 1.25)	-	1.18 (1.12 − 1.24)	5.3
Missing	1.36 (1.19 − 1.30)	1.39 (1.32 − 1.46)	1.24 (1.18 − 1.31)	38.5	1.25 (1.19 − 1.32)	-	1.23 (1.17 − 1.29)	4.2	1.24 (1.18 − 1.30)	-	1.22 (1.16 − 1.29)	8.3

*(The contribution of each factor was calculated as the percentage reduction in the OR of SES compared to Model 3 using the formula (OR Model 3 – OR Model A/B/C/D) / (OR Model 3 – 1).

Model 1 = Adjusted by SES.

Model 2 = Adjusted by SES + age and parity.

Model 3 = Adjusted by Model 2 + smoking.

Model A = Adjusted by Model 3 + place of residence grouped by hospital district.

Model B = Adjusted by Model 3 + major congenital anomaly.

Model C = Adjusted by Model 3 + amniocentesis.

Model D = Adjusted by Model 3 + amniocentesis + placenta previa + placental abruption + major congenital anomaly + prior cesarean section.

## Discussion

### Statement of principal findings

The aim of the present study was to identify risk factors for SGA among singleton live births and quantify their contribution to socioeconomic disparities in SGA. Out of the total population of singleton live births (*n* = 1,390,165) in Finland during the study period (1987–2010), 3.1% (*n* = 42,702) were classified as SGA, which corresponds well to a Swedish study that used the same definition of SGA and reported a 2.0% SGA incidence for the period 1999 to 2010 [13]. In the fully adjusted models, we detected a 11 − 24% higher SGA incidence in the lowest SES groups in comparison with the highest SES group, and an up to 1.5-fold difference in SGA incidence among the twenty hospital districts. The main finding of the present study was that smoking alone made the largest contribution, explaining about 40 − 50% of the excess risk of SGA among the lower SES groups. In contrast, the combined contribution of amniocentesis, placental abruption, placenta previa, congenital anomaly and prior CS was only around 4%.

### Strengths and weaknesses of the study

The most important strength of our study was that the data derived from the national, mandatory MBR covered the entire population, and therefore offered a comprehensive view of the risk factors for SGA. The MBR content is extensive, and thus allows the use of numerous birth characteristics, perinatal outcomes and socioeconomic factors as exposures. Further, to best of our knowledge, the present study population is one of the largest studied to date. On the other hand, this kind of register information might include errors and missing values because the data are produced mainly for administrative and statistical purposes rather than for research. However, the MBR has been shown to have excellent data coverage and quality [18,19]. A possible limitation of the study was that the gestational age was not estimated by ultrasonography for all women, which may have masked or indicated certain trends in the SGA rate across the time periods (because data for the earlier periods were more reliant on the last menstrual period without correction). Further, a possible limitation was that SES was determined by parturients’ occupation during pregnancy and we had no information on maternal educational or family income. In general, however, both of these variables are known to be related in our country to the occupation which in turn is a register-based, available indicator for studies on socioeconomic health differences in the perinatal period [10,20]. Spouses’ information was not available in the MBR due to the confidentiality of such information by the Finnish law.

### Meaning and implications of the study

Our results are in line with previous results and confirm that the etiology of SGA is multifactorial and substantially affected by SES [9]. After adjustment for background information, the risk of SGA was 11 − 24% higher in the lowest SES group than in the highest SES group. The risks of SGA and other adverse pregnancy outcomes have been shown to be affected by SES in multifarious ways. For example, exposure to harmful substances may result from occupational, residential and lifestyle factors that are socially patterned [9]. Our results revealed an up to 1.5-fold difference in SGA risk due to the parturient’s place of residence (after adjustment for background information). In the hospital districts with high SGA risk (districts 5, 6 and 16), the gross domestic product (GDP) per capita and unemployment rate have been reported to be among the lowest and highest in Finland, respectively (Local Finland, http://www.localfinland.fi/en/Pages/default.aspx). The main finding of the present study was that smoking alone made the largest contribution, explaining about 40 − 50% of the excess risk of SGA among the lowest SES groups. This implies that smoking during pregnancy resulted in 0.2 extra cases of SGA per 1,000 births in lower white-collar workers compared to 5.3 extra cases per 1,000 births in blue-collar workers. Among the total population, smoking during the first trimester and throughout the pregnancy resulted in 624 and 5850, respectively, additional cases of SGA during the study period. The apparently large contribution of smoking to socioeconomic disparities in SGA is in line with the results of a previous study that utilized data from the Finnish MBR for the years 1991 to 1999 [10]. The strong relation between smoking and birth weight is not a novel and unexpected finding, and the relationship has even shown to be dose-dependent [12]. However, our data did not contain information on the number of cigarettes smoked per day, and hence we did not examine the dose dependence. Nevertheless, the Finnish MBR has been shown to cover daily smoking habits during pregnancy relatively well [21]. Our results showed that women who quitted smoking during the first trimester of pregnancy had only a 7% higher risk of SGA to that of non-smokers, suggesting that interventional measures to reduce the socioeconomic gradient of SGA might prove effective. It could be argued that the inclusion of women (13.2%, *n* = 151,694) with missing information on SES may have biased the results. However, the incidence of SGA (3.4%) and proportions of women who gave up smoking (3.4%) or were smokers (14.1%) among the women with missing SES information were similar to those of the general population, showing that the results were unlikely to be biased by the missing information.

Our results also confirmed associations between SGA and several reproductive risk factors, such as nulliparity [6,7], advanced maternal age [6], BMI ≤24.9 [5,22], placental abruption [6,23], and congenital anomalies [24]. Furthermore, we found an association between amniocentesis or prior CS and SGA. Amniocentesis has previously been shown to be associated with an increased risk of pregnancy loss [25]. However, in our study, amniocentesis was probably a non-causal indicator of a high-risk pregnancy. Vaginal birth after prior CS (VBAC) has also been shown to be associated with other adverse maternal and neonatal outcomes [26,27]. Our results suggested that the combined contribution of amniocentesis, placenta previa, placental abruption, congenital anomaly and prior CS to the excess SGA risk among the lowest SES groups was up to 3.6%. However, we were not able to investigate all possible confounding factors found in previous studies, e.g., maternal chronic diseases, such as gestational hypertension [6] and preeclampsia [6] due to limitations in the availability and quality of data used. Furthermore, our results showed that the risk of SGA was inversely associated with gestational age, in particular, the incidence of SGA decreased by 41 − 52% after gestational week 40. This indicates that women with SGA were diagnosed and treated before term.

## Conclusions

We investigated the social risk profile of SGA infants (a birthweight below 2 SDs of the mean weight for gestational age) using data contained in a large 24-year register-based database covering the total population in Finland. Our results suggested that the etiology of SGA is multifactorial and substantially affected by SES and place of residence. After adjustment, an up to 1.5-fold difference in SGA risk was detected among the twenty hospital districts studied. The most important finding of the present study was that smoking served as a marker of lifestyle and explained about 50% of the excess risk of SGA in the lowest SES group in comparison with the highest SES group, causing in the order of 5.3 extra cases of SGA per 1,000 births over the study period. Smoking is a modifiable risk factor and our results showed that women who quitted smoking had a 7% higher risk of SGA to that of smokers. Thus, advocating smoking cessation for pregnant women and women attempting to become pregnant seems to be advisable.

## Competing interests

The authors declare that they have no competing interests.

## Authors’ contributions

All authors participated in designing the study (SR, MG, US, JS, MRK and SH). SR managed the dataset and performed statistical analyses. MG, US, JS, MRK and SH were statistical advisors. All authors contributed to the interpretation of the results, as well as to the writing and editing of the manuscript.
